# Ultrasensitive optical biosensor for detection of miRNA-155 using positively charged Au nanoparticles

**DOI:** 10.1038/s41598-018-20229-z

**Published:** 2018-02-13

**Authors:** Fatemeh Hakimian, Hedayatollah Ghourchian, Azam sadat Hashemi, Mohammad Reza Arastoo, Mohammad Behnam Rad

**Affiliations:** 1 Institute of Biochemistry and Biophysics, University of Tehran, Tehran, Iran; 2 Hematology, Oncology & Genetics Research Center, Shahid Sadoughi University of Medical Sciences, Yazd, Iran; 3 Department of Biological Sciences, Institute for Advanced Studies in Basic Sciences (IASBS), Zanjan, Iran

## Abstract

An ultrasensitive optical biosensor for microRNA-155 (miR-155) was developed to diagnose breast cancer at early stages. At first, the probe DNA covalently bind to the negatively charged gold nanoparticles (citrate-capped AuNPs). Then, the target miR-155 electrostatically adsorb onto the positively charged gold nanoparticles (polyethylenimine-capped AuNP) surface. Finally, by mixing citrate-capped AuNP/probe and polyethylenimine-capped AuNP/miR-155, hybridization occurs and the optical signal of the mixture give a measure to quantify the miR-155 content. The proposed biosensor is able to specify 3-base-pair mismatches and genomic DNA from target miR-155. The novelty of this biosensor is in its ability to trap the label-free target by its branched positively charged polyethylenimine. This method increases loading the target on the polyethylenimine-capped AuNPs’ surface. So, proposed sensor enables miR-155 detection at very low concentrations with the detection limit of 100 aM and a wide linear range from 100 aM to 100 fM.

## Introduction

Breast cancer is one of the main causes of cancer leading to death among women in the world^1,2^. Mammography and ultrasound scanning are the standard diagnostic techniques which have proven successful for early detection and diagnosis of breast cancer^2^. However, mammography has limited sensitivity, yielding a high rate of false-positive results. This approach may also bring about an accumulated exposure to radiation, which is considered an extra and crucial risk factor^3^. Ultrasound, on the other hand, is a non-invasive and safe method, but it cannot be replaced with mammography. Thus, it is thoughtful to develop a non-invasive, simple and low-risk method for screening or diagnosing breast cancer^4^. Among the various cancer biomarkers, microRNAs (miRs) have recently attracted researchers’ attention. These molecules are short endogenous molecules for noncoding ribonucleic acids that negatively regulate gene expression^5^. With the regulatory roles in gene expression, the abnormal expression of miRNAs is associated with the occurrence of different cancer types including breast cancer^6^. More specifically, there are several studies indicating the abnormal expression of miR-155 in breast cancer patients^7–9^. Therefore, overexpression of miR-155 suggests a risk factor for breast cancer^10^. Currently, methods for detection of miRNA mainly include reverse transcription polymerase chain reaction (RT-PCR)^11–13^, northern blotting^14,15^, microarray technique^16,17^, *in situ* hybridization^18–20^ and so on. Despite the advantages, these methods still suffer some disadvantages such as requiring expensive instruments and reagents, miRNA labeling, large amounts of miRNA and relatively pure miRNA samples^21^. Therefore, developing a rapid, simple and label free miRNA detection method is very important and highly desirable.

Colorimetric-based detection techniques have attracted great attention due to their simplicity, rapidness and low cost^22–24^. In colorimetric sensing methods, metallic nanoparticles, especially AuNPs because of their high extinction coefficient and strong distance- and size- dependent optical property^25^ are widely used^26–30^. The color of small AuNPs (~10–50 nm), when individually dispersed, is red due to the coherent oscillation of AuNP surface electrons (localized surface plasmon resonance)^31^. Upon aggregation, the solution color changes from red to pinkish/purple^32^ due to the coupling of AuNP surface plasmons^31,33^. In colorimetric-based biosensing assays based on AuNPs, there are two kinds of controlled aggregation of AuNPs: Interparticle bonding formation (interparticle crosslinking aggregation) and non-crosslinking aggregation that is produced by the removal of colloidal stabilization effects^34,35^.

The pioneers of the detection methods based on interparticle crosslinking aggregation of AuNPs were Mirkin and co-workers. They reported a novel method for colorimetric detection of DNA targets based on thiolated oligonucleotide modified GNPs (AuNP/probe) that used the distance-dependent optical properties of aggregated AuNP/probe. They used two sets of AuNP/probe with two different probes. After addition of a linker double-stranded DNA (dsDNA) with overhangs complementary to the two AuNP/probes, aggregation occurred and color of the solution changed from red to blue^36^. The same group one year later detected single-stranded DNA (ssDNA) with a detection limit of 10 fmol^32^. Thereinafter several analytes detected by using two kinds of probes that covalently attached to the surface of AuNPs^35,37–41^.

The biosensing assays based on non-crosslinking aggregation of AuNPs were reported for the first time by Maeda and co-workers^30^. In using this method several analytes were detected^42–44^. The main purpose of using colorimetric-based sensors being to improve sensitivity of such methods^24^. Several amplification techniques such as enzymatic signal amplification^45–47^, exonuclease-assisted signal amplification^48,49^ and rolling circle amplification^50–53^ have been developed to improve sensitivity of the colorimetric assays. But, these amplification methods have some disadvantages, such as requiring more time, higher costs and a more complex process^52^.

For the first time, in order to improve the sensitivity of biosensing assays based on crosslinking aggregation, branched polyethylenimine (PEI) capped AuNP (P-AuNP) were used for target trapping. P-AuNPs are highly positively charged because of the presence of nitrogen atoms of branched PEI, which makes them suitable for concentrating negatively charged molecules^54–56^ such as miR-155 resulting in the formation of P-AuNPs/miR-155 complex. On the other hand, thiolated hairpin probe DNA is covalently attached to the citrate-capped AuNPs (C-AuNPs) and the resulting C-AuNPs/probe. By mixing two complexes containing probe and target, DNA/RNA hybrid duplex is formed which cause interparticle cross-linking aggregation. This is quantified as an optical signal for monitoring the target. The significant and interesting point of this study is using branched PEI as concentrating miR-155 on it causes miR-155 detection at very low concentrations without the need for expensive and time-consuming signal amplifications.

## Results

### Sensing mechanism

**Figure 1:**
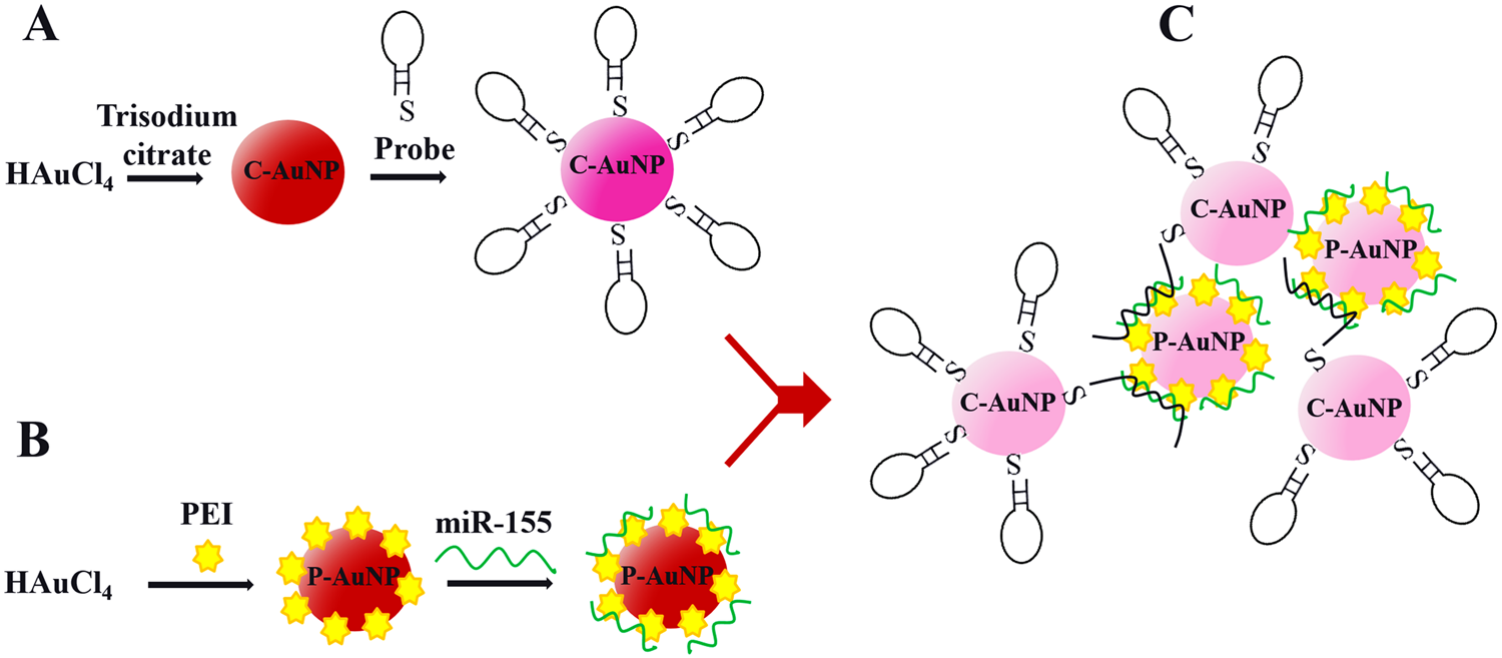
Nanoparticle aggregates, resulted from probe-target hybridization, enable optical detection of miR-155.

**Figure 2:**
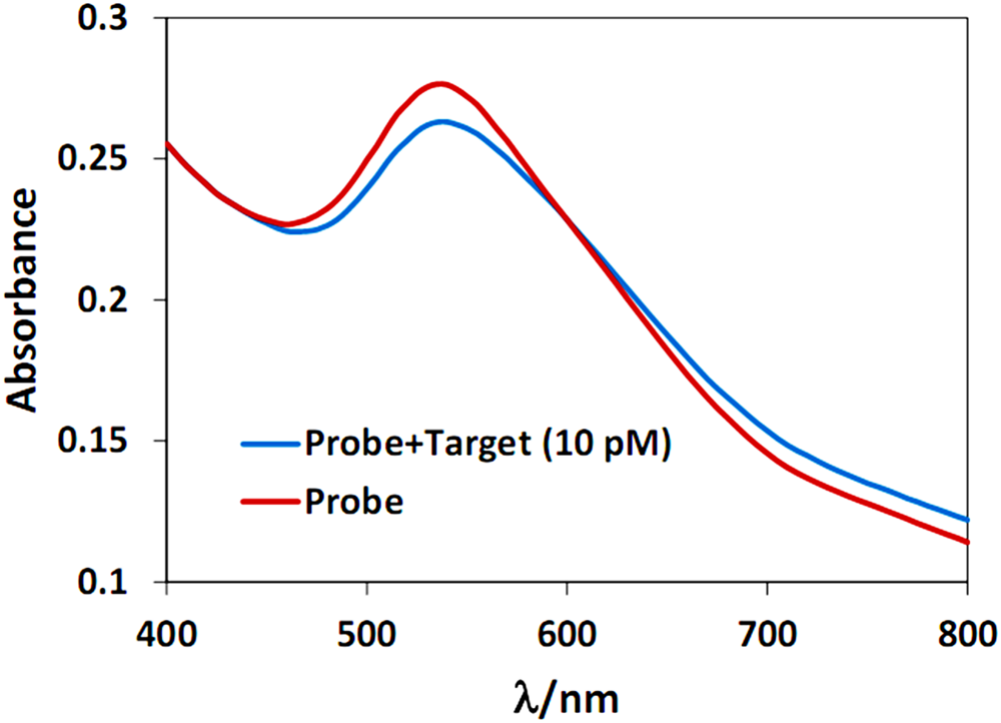
UV-Vis spectra of C-AuNP/probe + P-AuNP in the absence (red line) and presence (blue line) of miRNA-155.

Figure 1 illustrates the sensing mechanism of the proposed optical biosensor. In the first step, after C-AuNP synthesis, C-AuNPs are functionalized with the thiolated probes (Fig. 1A). It should be noted that thiolated probe molecules contain a nine-nucleotide thymidine spacer at its 3′ end which provides further optimal immobilization and hybridization efficiency^57^. In addition, the hairpin structure of our designed probe enhances its specificity more so than the corresponding linear ones^58^. In parallel, P-AuNPs are prepared and modified with miR-155 (Fig. 1B). Noteworthy, the color of C-AuNP/probe is dark pink while the color of P-AuNPs and P-AuNP/miR-155 are red. In the next step, when C-AuNP/probe add to P-AuNPs/miR-155, the mixture displays interparticle cross-linking aggregates; its color changes from red-pink to pink and the absorption intensity decreases at ~530 nm (Fig. 2). In fact, probe-target hybridization begins by addition of C-AuNP/probe to P-AuNP/miR-155, the distance between nanoparticles is reduced and, consequently, the aggregation process happens.

Since the UV-Vis absorption at ~530 nm indicates the quantity of dispersed nanoparticles and the absorbance at ~750 nm designates the aggregated AuNPs^44^, the absorbance ratio of 530/750 nm can express molar ratio of dispersed to aggregated AuNPs. Hence, the higher values of A_530_/A_750_, the greater stability of AuNPs and; the lower values of A_530_/A_750_, the greater aggregation.

### Characterization of C-AuNPs and P-AuNPs

**Figure 3:**
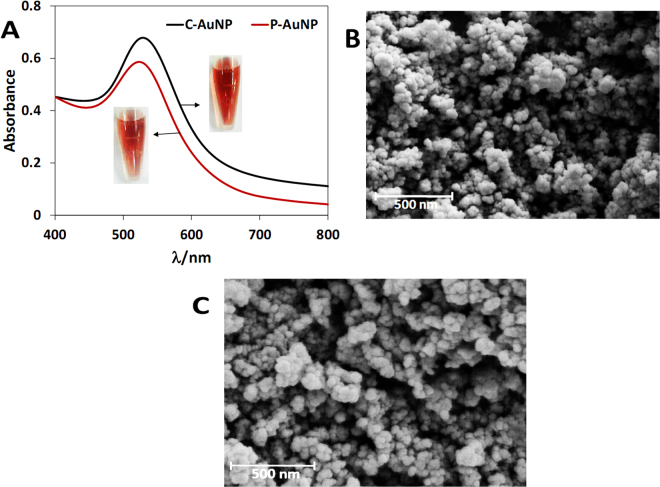
(**A**) UV−Vis absorption spectra of C-AuNPs and P-AuNPs. The inside images indicate colors of AuNPs solution. (**B** and **C**) SEM images of C-AuNPs and P-AuNPs, respectively.

Two types of gold nanoparticles were synthesized based on the method reported in the literature^59–61^ by slight modification. C-AuNPs and P-AuNPs were synthesized via reduction of HAuCl_4_ by using two reductants of trisodiumcitrate and polyethylenimine, respectively. Formation of C-AuNPs and P-AuNPs were confirmed by the UV-Vis spectra of two samples (Fig. 3A). The maxima of the plasmon resonance bands, located at ~530 nm, give evidence of the formation of C-AuNPs and P-AuNPs. The red color of the two samples (insets in Fig. 3A) demonstrates the successful formation of gold nanoparticles. SEM images also confirmed the formation of nanoparticles (Fig. 3B and C). Since citrate ions surround C-AuNPs and PEI cover P-AuNPs, these complexes are negatively and positively charged and their zeta potentials, as might be expected, have different signs i.e. −16.32 ± 0.17 mV and +36.51 ± 1.40 mV, respectively. In addition the hydrodynamic size of C-AuNPs and P-AuNPs were measured by DLS to be 21.2 ± 1.4 nm and 22.6 ± 0.3 nm, respectively (data not shown).

### Modification of C-AuNPs by thiolated probe and P-AuNPs by miR-155

**Figure 4:**
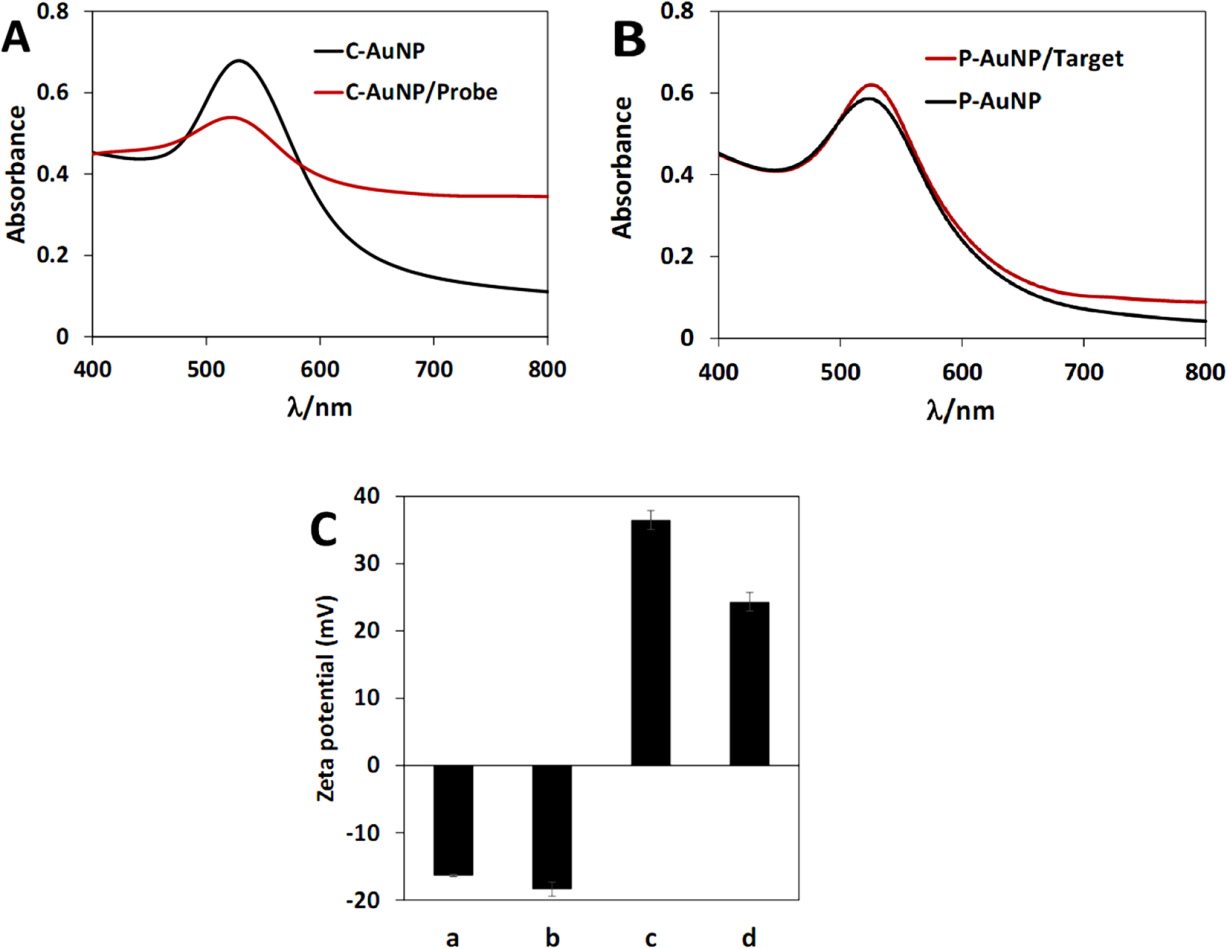
(**A**) UV-Vis spectra of C-AuNP and C-AuNP/probe, (**B**) UV-Vis spectra of P-AuNP and P-AuNP/target. (**C**) Comparing the zeta potential of a: C-AuNPs, b: C-AuNP/probe, c: P-AuNP and d: P-AuNP/target.

As mentioned in the previous section, attachment of thiolated probe molecules to C-AuNPs leads to the coverage of gold nanoparticles with probe molecules on the surface (C-AuNPs/probe) that are used for trapping target molecules. On the other hand, attachment of target molecules (miR-155) to the surface of P-AuNPs results in the formation of P-AuNPs/miR-155 with the ability of hybridization to the probe. To confirm the formation of P-AuNPs/miR-155 and C-AuNPs/probe, UV-Vis spectroscopy was used. As Fig. 4A shows, after coating of C-AuNPs with thiolated probes, the absorption at 530 nm decreases drastically, while that at 550 to 800 nm region increases and the color of nanoparticles changes from red to dark pink. As explained in the literature, the reason for changes in the position and intensity of absorption peak of gold nanoparticles is the changes of plasmon resonance frequency. Several factors influence these changes such as size and morphology of particles, dielectric constant of metal and environment^62^, shape and size of the charge distribution, electron density, effective electron mass^63^, electrolyte due to charge screening effects^64,65^ and agglomeration^66^ or aggregation of AuNPs. It seems that covering the C-AuNPs by the hairpin structured thiolated probes, possibly results in the vertical placement of probe on the surface of C-AuNPs. This may form a thick insulating layer on the surface of nanoparticles which causes some changes in the aforementioned physical properties of nanoparticles.

On the other hand, P-AuNPs possess positive charges. Therefore, they are able to bind to negatively charged phosphate groups of miR-155 through electrostatic interactions which results a slight decrease in the absorption at 600–800 nm (Fig. 4B). Mir-155 molecules are single stranded and may orient parallel to the nanoparticles’ surfaces and consequently form a weak thin insulation-film. This film, in turn, alters the physical surface properties of P-AuNPs including dielectric constant and surface plasmon resonance which causes the absorption change of P-AuNPs/mir-155 being minute relative to that of P-AuNPs alone.

To prove the successful attachment of probe to C-AuNP surface, the zeta potential of C-AuNPs before and after probe addition were compared. Reducing in zeta potential from −16.32 ± 0.17 to −18.35 ± 1.04 mV indicates successful attachment of probe molecules to C-AuNPs surface. The attachment of negatively charged probe molecules to nanoparticles increases the overall negative charges of the latter. On the other hand, after miR-155 addition, zeta potential of P-AuNPs decreases from +36.51 ± 1.40 to +24.34 ± 1.36 mV which implies negative charges of target molecules compensate positive charges of nanoparticles; an observation that confirms the successful attachment of miR-155 to P-AuNPs through electrostatic interactions.

### Calibration curve for detection of miR-155

Serial dilutions of miR-155 were prepared and analyzed based on the sensing mechanism (see Methods). To remove the background noise and therefore, better present the sensor response, we used Equation 1, in which $\delta {{\rm{A}}}_{(\frac{530}{750})}$ is a measure which indicates the amount of aggregated nanoparticles. The more concentration of miR-155, the more hybridization between probe and miR-155. This hybridization results in nanoparticles aggregation and therefore increment in $\delta {{\rm{A}}}_{(\frac{530}{750})}$ quantity. In Equation 1, ${{\rm{A}}}_{(\frac{530}{750})}$ indicates the absorption ratio of C-AuNP/probe +  P-AuNP/miR-155 at 530 nm to that at 750 nm. In addition, ${{\rm{A}}}_{(\frac{530}{750}){\rm{p}}}$ represents the absorption of C-AuNP/probe + P-AuNP at 530 nm to that at 750 nm.

1 $$\delta {{\rm{A}}}_{(\frac{530}{750})}=|\frac{{{\rm{A}}}_{(\frac{530}{750})}\,-{{\rm{A}}}_{(\frac{530}{750}){\rm{p}}}\,}{{{\rm{A}}}_{(\frac{530}{750})}}|=|\frac{{{\rm{\Delta }}{\rm{A}}}_{(\frac{530}{750})}\,}{{{\rm{A}}}_{(\frac{530}{750})}}|$$

**Figure 5:**
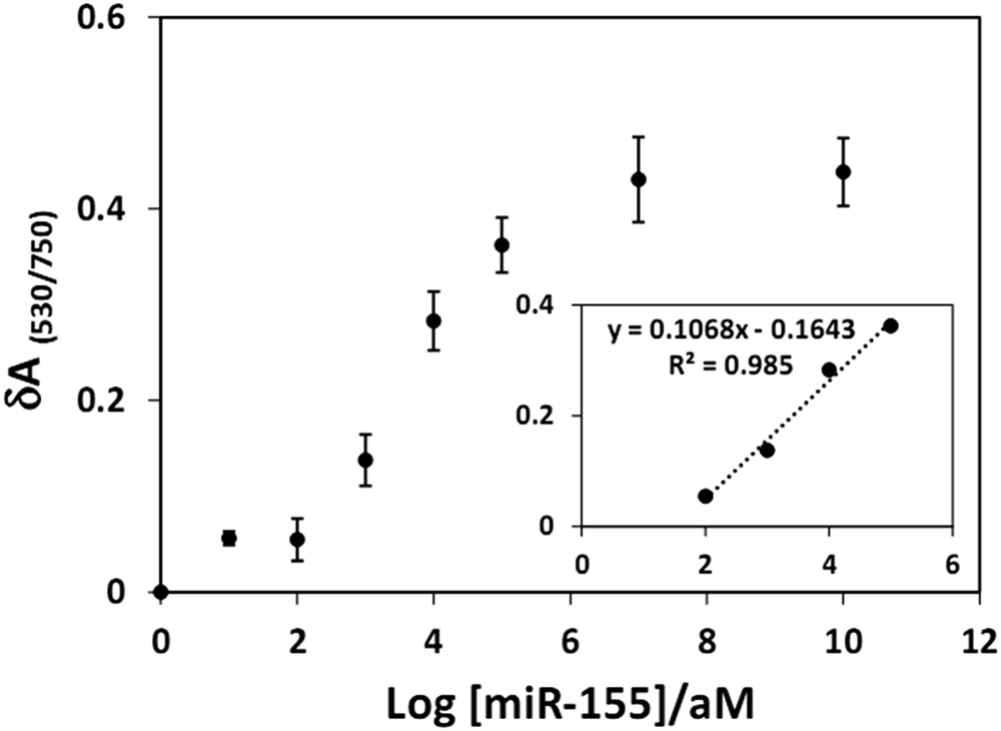
The $\delta {{\rm{A}}}_{(\frac{530}{750})}$ as a function of log (miRNA-155) concentrations. Each point stands for the mean value of three independent measurements. The inset shows the linear range of the calibration curve.

Based on the $\delta {{\rm{A}}}_{(\frac{530}{750})}$ vs. log miR-155 concentration, a calibration curve was plotted (Fig. 5). The linear segment of the resulting sigmoid from 10^2^ to 10^5^ attomolar (aM) was used as the calibration curve for quantitative detection of target (Fig. 5, inset). Upon increasing the concentration of miR-155, the number of dispersed nanoparticles descended while the number of aggregated nanoparticles ascended. Therefore, as shown in the calibration curve, the $\delta {{\rm{A}}}_{(\frac{530}{750})}$ value increases by increasing the target concentration. The intersection between two linear functions of maximum and minimum slope at low concentrations was used^59,67^ to estimate the limit of detection (LOD) of miR-155 to be 100 aM. As compared in Table 1, this LOD is much lower than those reported for other colorimetric methods^32,37,68–75^. Such a low LOD is probably due to the ability of the branched PEI to increase the target loading by concentrating^76^ miR-155 molecules on the P-AuNPs surface.

**Table 1:** Comparison between different colorimetric sensors for nucleic acid detection.

Strategy	Sensing mechanism	Target	detection limit	Assay time (min)	References
Assembly of thiolated probe/AuNP and PEI-AuNP/target	Interparticle crosslink	miRNA	100 aM	~60	Current method
Assembly of thiolated probe 1 and 2- AuNPs	Interparticle crosslink	DNA	10 fmol	~10	32
AuNPs assembly and ligase reaction-	Interparticle crosslink	DNA	74 pM	~120	37
AuNP & salt, PCR amplification	Non-crosslink	DNA	—	~120	68
AuNP & salt, PCR amplification	Non-crosslink	DNA	5 fmol	> 60	69
AuNP & salt, PCR amplification	Non-crosslink	DNA	6 μgml^−1^	~10	70
Temperature dependence of target triggered DNA-AuNP disassembly	Interparticle crosslink	DNA	300 nM	~10	71
Nicking endonuclease-assisted nanoparticle amplification	Interparticle crosslink	DNA	0.5 fmol	180<	72
AuNP & salt	Non-crosslink	DNA	4.3 nM	5	73
AuNP & conjugated Polyelectrolyte	Non-crosslink	DNA	1.25 pM	5–10	74
Multi-component AuNPs-probe/Magnetic microparticles-probe	Interparticle crosslink	DNA	25 pM	~180	75

### Selectivity of the optical biosensor

**Figure 6:**
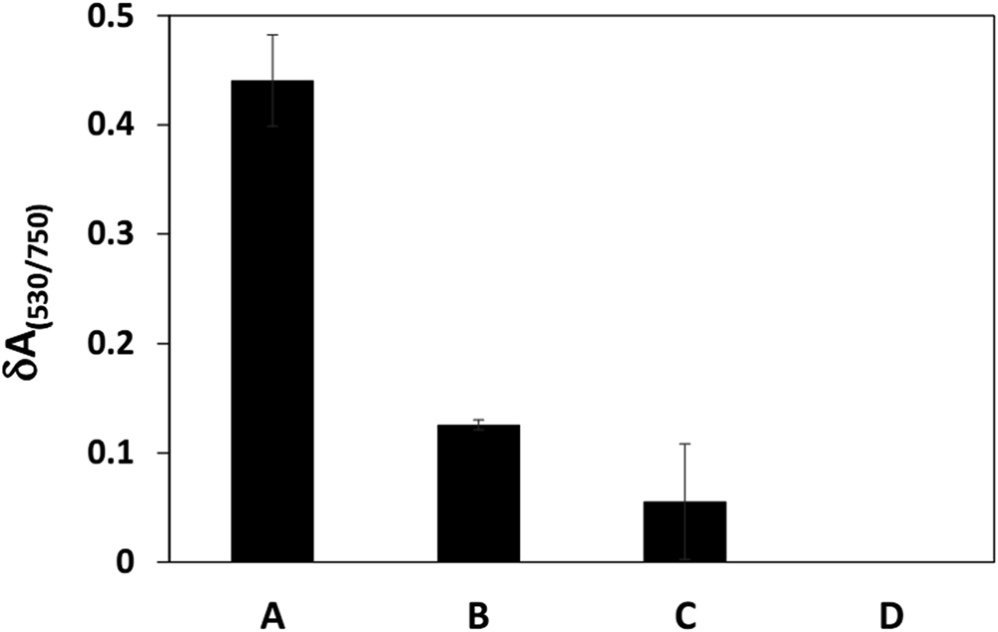
Selectivity of the optical biosensor toward miR-155 in comparison with different samples. **D:** C-AuNP/probe (Cp) + P-AuNPs, **A:** Cp + P-AuNPs/fully complementary miR-155, **B**: Cp + P-AuNPs/three base mismatched miR-155, and **C:** Cp + P-AuNP/genomic DNA.

The selectivity of the optical biosensor was studied using three sequences including perfectly complementary targets (miR-155), three-base mismatched strands and non-complementary strands (genomic DNA). Comparison between the three responses and background are shown in Fig. 6. As seen, the response toward perfectly complementary target is 3.5 times bigger than that of three-base mismatch sequence and about 8 times higher than that of the genomic DNA. The higher sensor response toward miR-155 indicate that the probe-target duplex formation is more probable in comparison to the other duplexes. To confirm this superiority in hybridization, we also calculated the hybridization free energy of probe-target and probe-three base mismatch sequence using “RNA structure” web server. Since, the stability and the probability of hybridization depend on the free energy of hybridization^77,78^, therefore it has been used for comparison between the stability of duplexes. Results showed that the hybridization energy of probe-target and probe-three base mismatch are −39 and −23/3 kJ/mol, respectively. Since, probe-target hybridization energy is more negative than that of the probe-three base mismatch, therefore, the probe-target duplex is more stable and probable. Indeed, free energy of hybridization is the sum of individual base-pairing reactions involved. Thus, the total number of complementary bases in the two strands determines the duplex stability and probability^78^. Since, probe-target has more base pair than probe-three base mismatch and probe-genomic DNA, we conclude that duplex stability and probability in probe-target is higher than those for other duplexes. Even there is a possibility of binding of each negatively charged molecule to P-AuNPs, only miR-155 binding causes more cross-linking aggregation.

## Discussion

A versatile, sensitive, simple, and cost-effective method for detection of miR-155 was developed for the first time. The sensing signal was based on the hybridization of probe/functionalized C-AuNPs and P-AuNPs modified with miR-155. The mechanism behind the designed sensor lies in the hybridization between probe and target i.e. miR-155 caused interparticle cross-linking aggregation of C-AuNPs and P-AuNPs; which in its macroscopic scale shows a change in color from red-pink to pink. The innovation of this method is the utilization of positively charged AuNPs that are modified with target by electrostatic interaction for interparticle cross-linking aggregation, this reduces time and steps of sensing along with no need to tag target. Moreover, by using the branched P-AuNPs, the absorption of miR-155 on the surface of nanoparticles increases. Of interest, this process dramatically improves the interparticle cross-linking aggregation and, consequently, the detection limit shifts to the lower values. This method can be used to detect any oligonucleotide simply by changing the probe sequence. We expect that sophisticated optimization of the ratio between C-AuNPs, probe, and P-AuNPs might lead to even higher sensitivity.

## Methods

### Chemicals

The HPLC purified oligonucleotide sequences are as follows: Hsa-miR-155 (5′-UUAAUGCUAAUCGUGAUAGGGGU-3′) and three-base mismatched hsa-miR-155 (5′-UUAAUGCUUAUCGAGAUACGGGU-3′) were purchased from Bioneer Corporation (Republic of South Korea). Thiolated miR-155 probe (5′-AAAAAAAAACCCCTATCACGATTAGCATTAATTTTTTTTT-HS-3′) was synthesized by AnaSpec, Inc. (Canada). HAuCl_4_, polyethylenimine (50% solution, M_n_ ~1200, M_w_ ~1300) and dialysis bag were obtained from Sigma-Aldrich (USA). Trisodiumcitratedihydrate (Na_3_C_6_H_5_O_7_.2H_2_O), HCl and Tween-20 were purchased from Merck (Germany).

### Preparation of C-AuNPs and C-AuNP/probe

C-AuNPs with the average diameter of 21 nm were synthesized by citrate reduction of HAuCl_4_^61^. In brief, 1.5 mL of sodium citrate 1% was added to 21 mL of boiling chloroauric acid solution 0.8 mM, while vigorously stirring until its color changed from pale yellow to deep red. The solution, then, was stirred for an additional 15 min and gradually cooled down to room temperature^61,79^. To prepare C-AuNP/probe, 400 µL C-AuNPs was mixed with 2 µL Tween 20 and 400 µL thiolated probe 1 µM, left for 48 hrs, and centrifuged for 23 min at 10,000 rpm. Finally, the supernatant was removed, and the oily red precipitate redispersed in 200 µL deionized water.

### Preparation of P-AuNPs and P-AuNP/miR-155

P-AuNPs were synthesized by thermal reduction with slight modification to the previous reports^59,60^. In brief, 100 µL of PEI 42 mM was added to 3 mL of 1.5 M HAuCl_4_ under vigorous stirring, pH adjusted to 7.4 with HCl, and then, the solution was brought to a boil; color change from yellow to red is an indication of the reduction process. The obtained nanoparticles were dialyzed against deionized water with a 3.5-kDa cutoff membrane. The resulting red solution was stored at 4 °C before use. For the preparation of P-AuNPs/miR-155, 5 µL of the miR-155 solution with specific concentration was incubated with 40 µL of synthesized P-AuNPs for 30 min at room temperature.

### Characterization

UV-Vis absorption spectra of the particle dispersions were measured using a Varian Cary Bio 100 spectrophotometer. The particles were characterized by dynamic light scattering (DLS) using a 90 Plus Pals (Brookhaven Instruments Corp., USA), and PALS zeta-particle sizing potential analyzer software. All optical sensing experiments were measured by a microplate reader (BioTek, PowerWave XS2, USA). Morphology and size of nanoparticles were further confirmed by scanning electron microscope (SEM) (KYKY, EM3200, China).

### Optical sensing of miR-155

For miR-155 detection, 40 µL of P-AuNPs and 5 µL of miR-155 solution were mixed and incubated for ∼30 min at room temperature, followed by the addition of 5 µL of C-AuNP/probe to it. After 15 min, UV-Vis absorption spectra of the aggregated particles were recorded by a microplate reader.
